# A retrospective investigation on clinical and radiographic outcomes of distal tibial fractures after intramedullary nailing using the lateral parapatellar extra-articular approach

**DOI:** 10.1007/s00402-024-05344-z

**Published:** 2024-04-23

**Authors:** Wei Nie, Zhaojun Wang, Shizhuang Xu, Sutong Guo, Yang Yue, Kefu Sun

**Affiliations:** Department of Orthopedic Surgery, Lianyungang 2nd People’s Hospital, No.41 East Hailian Road, Haizhou District, Lianyungang, Jiangsu Province China

**Keywords:** Distal tibial fracture, Semiextended, Lateral parapatellar extra-articular approach, Suprapatellar approach, Transpatellar approach, Intramedullary nail

## Abstract

**Introduction:**

According to reports, the modified extra-articular parapatellar approach allows the performance of tibial nailing in the semi-extended position without the concern of joint violation. However, there remains no special study that has provided a detailed assessment of the benefits and risks of this approach for treating distal tibial fractures (DTFs). The aim of this retrospective study was to investigate the clinical and radiological outcomes of patients with DTFs after intramedullary nailing using a lateral parapatellar extra-articular (LPE) approach in comparison to using the suprapatellar (SP) and transpatellar (TP) approaches.

**Methods:**

Data were collected from 99 patients with a minimum follow-up period of 12 months. Comparisons were conducted between the groups regarding the number of intraoperative fluoroscopies, complications, knee pain, knee range of motion (ROM), the Lysholm Knee Scale (LKS), the Olerud-Molander Ankle Score (OMAS) and radiological findings.

**Results:**

The demographic characteristics were comparable between the groups. Fewer intraoperative fluoroscopies were performed in the LPE (27.47 ± 4.98) and SP (26.03 ± 5.12) groups than in the TP group (30.20 ± 7.42; *P*<0.001). When compared with the other two approaches, the LPE approach was associated with less knee pain (*P*<0.001) and better knee ROM (*P*<0.001) at one week postoperative. No significant intergroup differences were detected in the incidence of complications, LKS scores (*P* = 0.687) and OMAS (*P* = 0.926). Radiological findings demonstrated that postoperative tibial alignment (*P* = 0.853), the time of bony union and rate of non-union were similar between the groups.

**Conclusion:**

The LPE approach can serve as a safe and effective option for tibial nailing, as it offers favourable outcomes in knee pain relief and knee ROM in the early postoperative period and is equivalent to the other two approaches in terms of the incidence of complications, fracture healing, functional recovery and postoperative alignment for patients with DTFs.

**Supplementary Information:**

The online version contains supplementary material available at 10.1007/s00402-024-05344-z.

## Introduction

Owing to the advantages of minimally invasive insertion and excellent biomechanical stability, the intramedullary nail (IMN) is recommended as a typical choice for the surgical treatment of most tibial shaft fractures. It can act as a useful tool for fracture reduction, particularly for fractures in the isthmal area [1]. Although current evolutions in nail design have expanded the application of IMNs to include the treatment of proximal and distal tibia fractures, it is still a challenge to treat these fractures with IMNs because of the broad medullary cavity and proximity of the fragments to the joint [2, 3]. The traditional transpatellar (TP) approach, which needs a hyperflexed position of the knee to access the nail starting portal, is closely correlated with angular malalignment and increased difficulty of intraoperative fluoroscopy. The current treatment strategy is in favor forthe semi-extended tibial nailing technique, and the suprapatellar (SP) approach has been commonly employed [4, 5]. Reportedly, nailing in a semi-extended position using the SP approach can facilitate fracture reduction, ease nail insertion, simplify the procedures of intraoperative fluoroscopies, and decrease the risk of malalignment, especially for patients with proximal tibial fractures [6]. Nevertheless, the intraarticular nature of the SP approach leads to concerns of joint infection, intraarticular debris, and iatrogenic damage to joint structures such as the patellofemoral cartilage, cruciate ligament, and anterior horn of the meniscus [7, 8]. Kubiak et al. [3] introduced a modified parapatellar approach that enables the extraarticular insertion of IMNs without the requirement for special instrumentation. Although some researches have been carried out on this approach, their conclusions were drawn based on patients with proximal tibial fractures or mixed types of fractures and no specific study exists which has focused on the outcomes of distal tibial fractures (DTFs) treated using this approach [8, 9]. We therefore conducted this study to evaluate the risks and benefits of the parapatellar extraarticular approach for treating DTFs by comparing the SP and TP approaches in terms of clinical and radiographic outcomes. As such, this study could be deemed as a supplement and an update to existing evidence.

## Methods

### Patient demographics

Data for this retrospective study were retrieved from the medical records of patients with DTFs who had undergone tibial nailing using one of these three approaches at our trauma centre from 2017 to 2021. The study was conducted in accordance with the standards of the Declaration of Helsinki and approved by the Ethics Committee of Lianyungang 2nd People’s Hospital (Ethics approval number: 2022-40). Patient signed informed consents were obtained accordingly for the publication of data and images included in this article.

DTFs were identified based on preoperative radiographic films and computed tomography scans and classified according to the Arbeitsgemeinschaft für Osteosynthesefragen/Orthopaedic Trauma Association (AO/OTA) classification system [10]. Patients who were diagnosed with extraarticular distal tibial fractures (OTA 43-A) with or without an extension of the nondisplaced interarticular fracture line (OTA 43-C1) were selected. The exclusion criteria included patients aged less than 18 years, those with pathological fractures, open fractures of Gustilo-Anderson type III, ipsilateral femoral fractures, concomitant severe neurovascular injury, preexisting knee or ankle arthritis or previous tibial deformity, and those lost to follow-up.

**Fig. 1:**
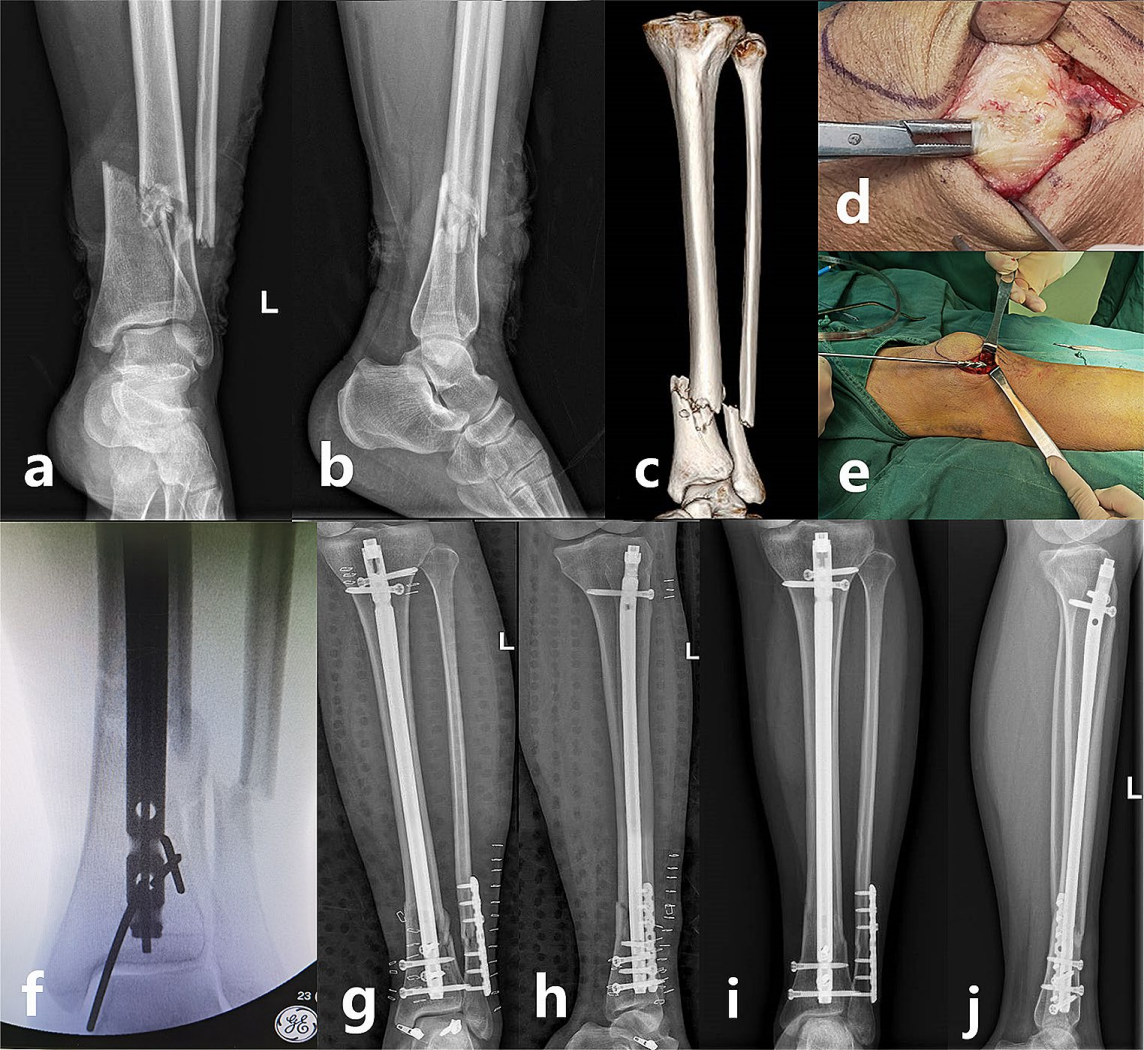
Preoperative anteroposterior (**a**) and lateral (**b**) plain radiographs and the 3-dimensional reconstruction CT scan (**c**) were taken for a 38-year-old man who sustained a distal tibial fracture and a concomitant fibular fracture in the left side. He experienced the semi-extended tibial nailing through the lateral parapatellar extraarticular approach. The intraoperative photograph shows that the retinaculum was separated from the underling joint capsule (**d**). A rigid handled reamers was used to drill the proximal metaphysis so as to keep the right alignment of reaming (**e**). K-wires were used as blocking screws for fracture reduction (**f**). Satisfactory reduction was confirmed on the postoperative plain radiographs (**g**, **h**) two days after surgery. Bony union was noted on the plain radiographs (**i**, **j**) at the 5-month follow-up

After screening, we identified 131 patients with DTFs treated with IMNs. Among them, 99 patients who met the inclusive criterion were involved in this study. Thirty-two of them were treated using a lateral parapatellar extraarticular (LPE) approach (the parapatellar approach was routinely performed through a lateral incision in our centre except in cases in which there was problem with the soft tissue or the medial mobility of the patella), 30 were treated with the SP approach, and the rest were treated with the TP approach. All patients were operated on by the same surgical team using the Expert Tibial Nail® (Trauson, Changzhou, China). During surgery, assistive techniques for fracture reduction, such as blocking screws, percutaneous clamps and minimally invasive wiring technique, were used if necessary (Fig. 1). Fixation of fibular fracture would be reserved when there was obvious malalignment or associated ankle instability.

### Postoperative management and follow-ups

Standard anteroposterior and lateral radiographs were taken within two days postoperatively. The duration of intravenous antibiotics was administered for less than 48 h for patients with closed fractures and was appropriately extended for patients with open fractures. Early active motions and partial weight-bearing with or without crutches were encouraged as tolerated. Follow-ups were scheduled for one week after surgery, as well as one, three, six, and 12 months postoperatively.

### Outcome measures

Assessments were mainly based on clinical and radiographic outcomes. Comparisons of clinical outcome were conducted between groups in terms of operative time, number of intraoperative fluoroscopies (only during tibial nailing), knee pain severity, knee range of motion (ROM), postoperative complications, and functional assessments of both the knee and ankle. The number of intraoperative fluoroscopies was collected from the computer of the C-arm fluoroscope (General Electric Healthcare, Chicago, United States). Knee pain was estimated via the numerical rating scale (NRS), in which zero means “painless” and ten indicates “worst possible pain”. Functions of the knee and ankle were evaluated at the 12-month follow-up using the Lysholm Knee Scale (LKS) [11] and the Olerud-Molander Ankle Score (OMAS) [12], respectively.

Radiographic evaluations included postoperative alignment and the status of bony union. Postoperative alignment was determined by the lateral distal tibia angle (LDTA) and the anterior distal tibia angle (ADTA), which were measured on anteroposterior and lateral radiographs based upon Paley’s method [13] and were judged according to the criterion established by Beebe et al. [14]. If the ADTAs and LDTAs fell within the normal ranges for the LDTAs (89 ± 3 degrees) and the ADTAs (80 ± 2 degrees), the result would be considered “excellent”. When those were within five degrees of the normal ranges, they were deemed “acceptable”. Otherwise, a deviation of more than five degrees from the normal range of values was judged as malalignment. X-ray photographs were taken at monthly intervals before bony union, which was defined as the appearance of bony continuity on at least three out of four cortices on radiographs. Fracture healing that exceeded six months was deemed delayed union. Nonunion was considered as the absence of bridging callus beyond nine months postoperatively and no sign of progressive callus formation throughout the past three months.

### Statistical analyses

Normally distributed continuous data were presented in the form of the mean ± standard deviation (SD) and were compared by one-way ANOVA, with post hoc comparisons using the least-significant difference method. Nonnormally distributed continuous data were presented as the median (interquartile range, IQR) and were compared by the nonparametric Kruskal-Wallis *H* test with post hoc comparisons using Bonferroni correction. Categorical data were reported as counts, and the related associations were determined by the Chi-square test or Fisher-Freeman-Halton test. All analyses were performed using SPSS 27.0 (Chicago, IL, USA), with a *P* value less than 0.05 set as statistically significant.

## Results

### Characteristics of patient demographics

The demographic characteristics of the patients are summarized in Table 1. Baseline data of the patients were comparable between the three groups.

**Table 1:** Demographic data of the patients

	LPE group	SP group	IP group	Statistical value	P
No. of patients	32	30	37	-	-
Age	42.19±12.26	40.37±10.46	43.49±10.91	0.640	0.530^a^
Sex					
Male/ Female	19/13	20/10	22/15	0.464	0.840^b^
Mechanism of injury					
Traffic accident	14	10	14	0.927	0.935^c^
Falling	6	8	9		
Crush injury	7	9	10		
Sport related injury	5	3	4		
Open fracture					
Gustilo-Anderson I	5	3	7	1.476	0.857^c^
Gustilo-Anderson II	4	3	3		
Interarticular extension of fracture line	2	3	5	0.992	0.590^c^
Ipsilateral fibular fractures	22	19	27	0.225	0.898^b^
Fixation to the fibula	11	8	13	0.634	0.741^b^
Chronic disease					
Hypertension	5	3	7	1.028	0.607^c^
Diabetes	3	4	6	0.732	0.704^c^
Follow-up period	20.44±4.74	20.83±5.30	22.49±5.34	1.562	0.215^a^

*Note*^a^: One-Way ANOVA analysis; ^b^: Chi-square test; ^c^: Fisher-Freeman-Halton exact test

### Clinical outcomes

The number of intraoperative fluoroscopies during tibial nailing was significantly higher in the TP group (30.20 ± 7.42) than in the LPE (27.47 ± 4.98) and SP (26.03 ± 5.12) groups (*F* = 27.896, *P* < 0.001). However, no significantly statistical difference was noted between the latter two groups (*P* = 0.346). The surgical time of TP (86 (73–117) mins) group was significantly longer than that of the LPE (68 (57–103) mins) and SP (65 (60–93) mins; *P* = 0.002) groups (Table 2).

**Table 2:** The comparison of outcomes between three groups

	LPE group	SP group	TP group	Statistic value	P
Numbers of intraoperative fluoroscopies (mean±SD)	27.47±4.98	26.03±5.12	30.20±7.42	27.896	Total: <0.001^a^ LPE vs. SP: 0.346^b^ LPE vs. TP: <0.001^b^ SP vs. TP: <0.001^b^
Operative time (min) (median, IQR)	68(57-103)	65 (60-93)	86 (73-117)	12.290	Total: 0.002 ^c^ LPE vs. SP: 1.000^d^ LPE vs. TP: 0.010^d^ SP vs. TP: 0.007^d^
Angulation					
Excellent	23	23	25	1.508	0.853^e^
Acceptable	7	4	8		
Malalignment	2	3	4		
Functional recovery (mean±SD)					
LKS	92.09±5.64	90.93±5.87	91.81±5.00	0.377	0.687^a^
OMAS	92.66±6.22	92.17±6.91	92.03±7.40	0.077	0.926^a^
Complications					
Thrombosis	2	1	2	0.565	0.866^e^
Delayed union	3	3	5	0.400	0.852^e^
Nonunion	1	0	2	1.488	0.774^e^
Time of union (median, IQR)	4.5 (4-5)	4.5 (4-6)	4 (4-6)	0.611	0.737^b^

*Note*^a^: One-Way ANOVA analysis; ^b^: post-hoc comparisons based on least-significance difference method; ^c^: non-parametric test based on Kruskal-Wallis H method; ^d^: post-hoc comparisons using Bonferroni correction; ^e^: Fisher-Freeman-Halton exact test; LKS: Lysholm Knee Scale; OMAS: Olerud-Molander Ankle Score

At one week postoperatively, the LPE group had lower NRS scores, and the differences were statistically significant between the LPE and SP groups (*P* = 0.008) as well as between the LPE and TP groups (*P* = 0.001), but not between the SP and TP groups (*P* = 1.000) (Table 2). However, this difference was insignificant at subsequent follow-ups. At 12 months after the operation, approximately 73% of patients reported being pain-free during routine daily life and activities. The remaining patients reported only mild pain without significant limitations in daily living, except for three patients who complained of moderate pain when climbing stairs and squatting, which was mainly caused by nail prominence and alleviated after the second operation for hardware removal.

**Fig. 2:**
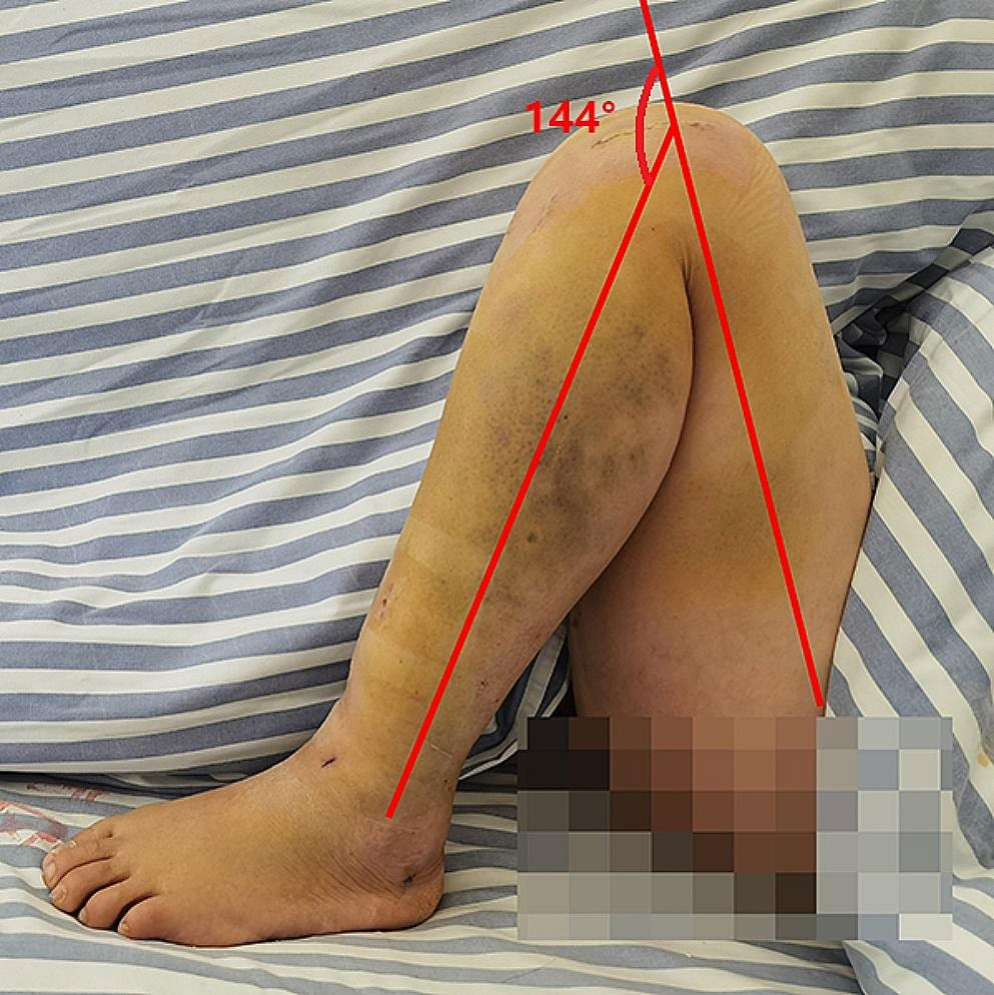
A 42-year-old female patient treated with the lateral parapatellar extraarticular approach demonstrated the encouraging outcome of knee flexion (144 degrees) at one week postoperatively

The recovery of knee ROM showed a similar changing trend with the decrease of knee pain severity. At one week postoperatively, the mean value of knee ROM was 103.63±9.15 degrees in the LPE group (Figs. 2), 75.83±9.66 degrees in the SP group and 72.95±9.26 degrees in the TP group (*F* = 116.600, *P*<0.001). As time progressed, the differences became less pronounced, and the knee ROM did not differ significantly between groups at one month (*F* = 2.815, *P* = 0.065), three months (*F* = 0.434, *P* = 0.649), six months (*F* = 0.294, *P* = 0.746) and 12 months (*F* = 0.034, *P* = 0.966) (Table 2).

No sign of surgery-related infection was observed. Asymptomatic distal deep vein thrombosis (DVT) was identified by ultrasound in five patients, which only needed immobilization and enhanced anticoagulant therapy (Table 3). At the last follow-up, there were no obvious signs of patellar instability, such as patella tilt, subluxation or dislocation, in any of the patients.

**Table 3:** The outcomes of knee NRS and ROM between three groups

	Inspection date	Groups			Compared groups	Statistical value	P value
		LPE group	SP group	IP group			
Knee ROM	1 week	103.63±9.15	75.83±9.66	72.95±9.26	Total	116.6	<0.001^a^
(Mean±SD)					LPE vs. SP		<0.001^b^
					LPE vs. TP		<0.001^b^
					SP vs. TP		0.194^b^
	1 month	111.27±13.95	102.71±10.54	100.68±8.48	Total	2.815	0.065^a^
	3 months	121.32±9.93	118.29±10.15	115.89±9.93	Total	0.434	0.649^a^
	6 months	127.00±10.42	126.41±10.37	123.74±10.74	Total	0.294	0.746^a^
	12 months	129.63±9.87	130.18±11.04	127.74±11.52	Total	0.034	0.966^a^
knee NRS	1 week	2 (1-4)	4 (3, 5)	4 (4, 5)	Total	15.306	<0.001^c^
(Median, IOR)					LPE vs. SP	-21.620	0.008^d^
					LPE vs. TP	-25.156	0.001^d^
					SP vs. TP	-3.536	1.000^d^
	1 month	2 (0.25-3.75)	2 (0.75-4)	2 (1-4)	Total	0.369	0.831^c^
	3 months	0 (0-2.75)	1 (1-3)	1 (0-4)	Total	0.334	0.846^c^
	6 months	0 (0-1)	0 (0-2)	0 (0-3)	Total	0.939	0.625^c^
	12 months	0 (0-0.75)	0 (0-1)	0 (0-1)	Total	0.685	0.710^c^

*Note*^a^: One-Way ANOVA analysis; ^b^: post-hoc comparisons based on least-significance difference method; ^c^: non-parametric test based on Kruskal-Wallis H method; ^d^: post-hoc comparisons based on Bonferroni correction; NRS: numerical rating scale; ROM: range of motion

On functional assessment at 12 months, the LKS score (*F* = 0.377, *P* = 0.687) and OMAS (*F* = 0.077, *P* = 0.926) indicated that there were no significant differences in the functions of the knee and ankle (Table 3). Most patients were satisfied with their clinical and functional outcomes.

### Radiographic assessments

Postoperative alignment was deemed excellent in 71 patients (23 in the LPE group (71.8%), 23 in the SP group (76.7%) and 25 in the TP group (67.5%)), acceptable in 19 patients (7 in the LPE group (21.9%), 4 in the SP group (13.3%) and 8 in the TP group (21.6%)), and malalignment in 9 patients (2 in the LPE group (6.3%) and 3 in the SP group (10.0%) and 4 in the TP group (10.8)). No marked intergroup difference was noted regarding the overall distribution of the postoperative alignment levels (*P* = 0.853) (Table 3).

Radiological manifestations demonstrated no significant difference between the three approaches in the time to radiological union (*P* = 0.737) and the rates of delayed union (*P* = 0.852) and nonunion (*P* = 0.774). A fracture healing time exceeding six months was observed in 14 patients. For 3 of them, the 6-month radiographs showed the processes of callus formation, and bony union was achieved when the observation period was prolonged to nine months. Eight patients were treated with nail dynamization and eventually healed. The remaining three patients failed to achieve bony union and required reoperations.

## Discussion

The benefits of semi-extended tibial nailing have been well documented [4–6]. A semi-extended knee can mitigate the deforming forces caused by the patellar tendon when the knee is in hyperflexion, which may be especially relevant to proximal tibia fractures. A horizontally placed lower leg will undoubtedly facilitate fracture reduction and intraoperative fluoroscopy, therefore decreasing the radiation time and exposure, which has been observed in the present and previous studies [15]. It should be noted that the data of surgical time in this study were reviewed from surgical records, which just provided the total operative duration. Given that many patients underwent both the tibial nailing and fibular fixation, this result could not be deemed as an accurate evaluation on the time spending on tibial nailing.

A proposed advantage of the semi-extended tibial nailing technique is the improvement in post nailing alignment [6, 7]. Avilucea et al. [6] observed that angular malalignment of more than five degrees after tibial nailing for DTFs occurred in 35 (26.1%) patients in the infrapatellar group but in only five (3.8%) patients in the suprapatellar group. We, however, did not find meaningful intergroup differences in either post nailing alignment or subsequent limb function in this study. Our result is similar to that of an investigation by Bakhsh et al. [16], who reported no obvious association between surgical approaches (parapatellar, suprapatellar, and infrapatellar) and postoperative angulation. A possible explanation is that the reduction of distal tibial fractures is easier than that of proximal fractures since the deforming force on distal tibial fragments caused by hyperflexion is not as powerful as that on proximal tibial fragments. In addition, the applications of intraoperative fluoroscopy and additional reduction techniques, including minimal invasive wiring technique, blocking screws and percutaneous clamps, will guarantee an accurate reduction.

Whether surgical approaches could have impacts on postoperative anterior knee pain is still the subject of ongoing debate [5, 17]. The exact aetiology of post nailing knee pain remains unconfirmed and may be multifactorial, which may relate to implant prominence, dissection to the patellar tendon, transsection of the inferior branches of the saphenous nerve, damage to the cartilage and detriments to infrapatellar fat pads [9, 18–20]. Moreover, many studies found the shorten of patellar tendon and the decrease of the Insall-Salvati index after tibial nailing using the transpatellar approaches. But they were in disagreement over whether these changes are associated with increased knee pain and worse functional outcomes [21, 22]. Being a completely extraarticular approach, the modified parapatellar approach is believed to avoid the damages to the aforementioned intraarticular structures and the deposition of debris, thereby minimizing physiological and functional disturbances to the knee [3]. Additionally, in order to avoid saphenous nerve transection, a lateral approach is preferred over the medial approach in our centre. We observed significant improvement in knee ROM and pain relief in the LPE group at one week postoperatively, which has not been reported in previous studies. These findings supported the hypothesis that the aforementioned advantages of the LPE approach might contribute to the prevention of functional disturbances on the knee. Interestingly, at one month and thereafter, these results were almost identical between groups, as well as the functional scores at 12 months postoperatively.

As an alternative to the SP approach, the parapatellar extraarticular approach can be applied to patients with patellofemoral arthritis that have a narrow joint space, which is supposed to be a contraindication for the SP approach. The feasibility of this approach has been proved in a study conducted by Patel et al., in which the LPE approach could be successful accomplished by surgeons who were not special trained [23]. According to our experience, most surgeons can perform this approach proficiently after 3 to 4 times of practices. Furthermore, the parapatellar approach can be extended to resolve periarticular fractures of the knee joint (see Supplementary Material 1). However, this approach is not free from complications. Because the entry point is prone to be lateral and posterior in the LPE approach, there is a possibility of lateral plateau cartilage violation and nail malposition [9, 24]. Fluoroscopic confirmation of the ideal entry point is of critical importance to avoid these complications [25]. This approach is also associated with the risk of capsular rupture. To minimize this risk, Kubiak et al. [3] recommended sufficiently releasing the retinaculum and providing adequate irrigation during reaming or nailing. Another concern is the potential of patellar instability. However, it has not been reported in existing evidence. Lu et al. [26] demonstrated another semi-extended nailing technique using an infrapatellar extraarticular approach that does not require the dissection of the retinaculum, thus eliminating the chance of patellar instability. Nonetheless, nailing using this approach requires a newly designed intramedullary nail and special instrument, which might limit the routine use of this approach.

There were a few limitations in the present study. Selection bias is inevitable due to its retrospective nature. Many patients asked for another operation for hardware removal. In some patients, the tibial nail was placed using the SP approach but was removed via an infrapatellar approach. That would interfere with the long-term assessment. Therefore, the duration of follow-up period was limited to 12 months and longer-term outcomes have not been evaluated in this study. However, to assess most of the parameters in this study, we believe that 12 months might be an appropriate and pragmatic follow-up period after tibial nailing. Additionally, cartilage injury of the patella-femoral joint and intraarticular debris are supposed to be important complications of the SP approach. As MR and arthroscopy examinations are not routinely performed for patients with DTFs in our centre, we could not accurately identify the existence of these complications. Given these, additional rigorous studies, randomized control trials or otherwise, are still needed to address the aforementioned limitations.

## Conclusions

Evidence from the present study, as observed in 99 patients with DTFs, suggests that the LPE approach, as well as the SP approach, can simplify surgical procedures, decrease the number of intraoperative fluoroscopies and reduce surgical time. However, there is no difference in radiographic performance and clinical outcomes including postoperative complications, functional scores and bony healing between any groups, with the exception of a significant but slight improvement in knee ROM and pain relief in the LPE group at the early postoperative period.

### Electronic supplementary material

Below is the link to the electronic supplementary material.

Supplementary Material 1

## Data Availability

The datasets used and/or analyzed during the current study are available from the corresponding author on reasonable request.

## Abbreviations

ADTA: Anterior distal tibia angle
ANOVA: Analysis of Variance
AO/OTA: Arbeitsgemeinschaft für Osteosynthesefragen/Orthopaedic Trauma Association
DTF: Distal tibial fracture
IMN: Intramedullary nail
IQR: Interquartile range
LDTA: lateral distal tibia angle
LKS: Lysholm Knee Scale
LPE: Lateral parapatellar extra-articular
OMAS: Olerud-Molander Ankle Score
ROM: Range of motion
SD: standard deviation
SP: suprapatellar
TP: transpatellar

## References

[1] Duan X, Al-Qwbani M, Zeng Y, Zhang W, Xiang Z (2012). Intramedullary nailing for tibial shaft fractures in adults. Cochrane Database Syst Rev.

[2] Chun DI, Min TH, Kang EM, Yu W, Won SH, Cho J (2022). Comparison of Radiological and clinical outcomes in patients treated with standard plating versus Intramedullary Nailing in Distal Tibial fracture. Orthop Surg.

[3] Kubiak EN, Widmer BJ, Horwitz DS (2010). Extra-articular technique for semiextended tibial nailing. J Orthop Trauma.

[4] Chan DS, Serrano-Riera R, Griffing R, Steverson B, Infante A, Watson D (2015). Suprapatellar Versus Infrapatellar tibial nail insertion: a prospective Randomized Control Pilot Study. J Orthop Trauma.

[5] Weil YA, Gardner MJ, Boraiah S, Helfet DL, Lorich DG (2009). Anterior knee pain following the lateral parapatellar approach for tibial nailing. Arch Orthop Trauma Surg.

[6] Avilucea FR, Triantafillou K, Whiting PS, Perez EA, Mir HR (2016). Suprapatellar Intramedullary nail technique lowers rate of Malalignment of Distal Tibia fractures. J Orthop Trauma.

[7] Jones M, Parry M, Whitehouse M, Mitchell S (2014) Radiologic outcome and patient-reported function after intramedullary nailing: a comparison of the retropatellar and infrapatellar approach. J Orthop Trauma 28(5):256 –262. 10.1097/BOT.0000000000000070 10.1097/BOT.0000000000000070 24464093

[8] Zamora R, Wright C, Short A, Seligson D (2016). Comparison between suprapatellar and parapatellar approaches for intramedullary nailing of the tibia. Cadaveric Study Injury.

[9] Baker HP, Strelzow J, Dillman D (2022). Tibial alignment following intramedullary nailing via three approaches. Eur J Orthop Surg Traumatol.

[10] Meinberg EG, Agel J, Roberts CS, Karam MD, Kellam JF (2018). Fracture and dislocation classification Compendium-2018. J Orthop Trauma.

[11] Tegner Y, Lysholm J (1985). Rating systems in the evaluation of knee ligament injuries. Clin Orthop Relat Res.

[12] Olerud C, Molander H (1984). A scoring for symptom evaluation after ankle fracture. Arch Orthop Trauma Surg.

[13] Paley D, Paley D (2002). Normal lower limb alignment and joint orientation. Principles of deformity correction.

[14] Beebe MJ, Morwood M, Serrano R, Quade JH, Auston DA, Watson DT (2019). Extreme Nailing: is it safe to allow Immediate Weightbearing after Intramedullary nail fixation of extra-articular distal tibial fractures (OTA/AO 43-A)?. J Orthop Trauma.

[15] Al-Azzawi M, Davenport D, Shah Z, Khakha R, Afsharpad A (2021). Suprapatellar versus infrapatellar nailing for tibial shaft fractures: a comparison of surgical and clinical outcomes between two approaches. J Clin Orthop Trauma.

[16] Bakhsh WR, Cherney SM, McAndrew CM, Ricci WM, Gardner MJ (2016) Surgical approaches to intramedullary nailing of the tibia: Comparative analysis of knee pain and functional outcomes. Injury 47:958 – 961. 10.1016/j.injury.2015.12.025 10.1016/j.injury.2015.12.025 26830120

[17] Keating JF, Orfaly R, O’Brien PJ (1997). Knee pain after tibial nailing. J Orthop Trauma.

[18] Bhattacharyya T, Seng K, Nassif NA, Freedman I (2006). Knee pain after tibial nailing: the role of nail prominence. Clin Orthop Relat Res.

[19] Leliveld MS, Verhofstad MH (2012) Injury to the infrapatellar branch of the saphenous nerve, a possible cause for anterior knee pain after tibial nailing? Injury 43:779 –783. 10.1016/j.injury.2011.09.002 10.1016/j.injury.2011.09.002 21962297

[20] Tornetta P 3rd, Riina J, Geller J, Purban W (1999) Intraarticular anatomic risks of tibial nailing. J Orthop Trauma 13(4):247 – 251. 10.1097/00005131-199905000-00004 10.1097/00005131-199905000-00004 10342349

[21] Graulich T, Gerhardy J, Omar Pacha T, Örgel M, Macke C, Krettek C, Omar M, Liodakis E (2022). Patella baja after intramedullary nailing of tibial fractures, using an infrapatellar/transtendinous approach, predicts worse patient reported outcome. Eur J Trauma Emerg Surg.

[22] Turkmen I, Saglam Y, Turkmensoy F, Kemah B, Kara A, Unay K (2017). Influence of sagittal plane malpositioning of the patella on anterior knee pain after tibia intramedullary nailing. Eur J Orthop Surg Traumatol.

[23] Patel AH, Wilder JH, Lee OC, Ross AJ, Vemulapalli KC, Gladden PB (2022). A review of proximal tibia entry points for Intramedullary Nailing and Validation of the lateral Parapatellar Approach as Extra-articular. Orthop Rev (Pavia).

[24] Hernigou P, Cohen D (2000). Proximal entry for intramedullary nailing of the tibia. The risk of unrecognised articular damage. J Bone Joint Surg Br.

[25] Althausen PL, Neiman R, Finkemeier CG, Olson SA (2002 Nov-Dec) Incision placement for intramedullary tibial nailing: an anatomic study. J Orthop Trauma 16(10):687–690. 10.1097/00005131-200211000-00001 10.1097/00005131-200211000-00001 12439190

[26] Lu K, Wu ZQ, Wang HZ, Qian RX, Li C, Gao YJ (2022). The semi-extended infrapatellar intramedullary nailing of distal tibia fractures: a randomized clinical trial. J Orthop Traumatol.

