# Supplementary material for: A retrospective investigation on clinical and radiographic outcomes of distal tibial fractures after intramedullary nailing using the lateral parapatellar extra-articular approach
Source: Arch Orthop Trauma Surg. 2024 Apr 23;144(5):2101–8. doi: 10.1007/s00402-024-05344-z (PMC11093779; doi:10.1007/s00402-024-05344-z)
Supplement: Supplementary file 1 — Supplementary Material 1 [file 402_2024_5344_MOESM1_ESM.pdf]

Supplementary Material 1. The application of the extended LPE approach used for periarticular fractures.

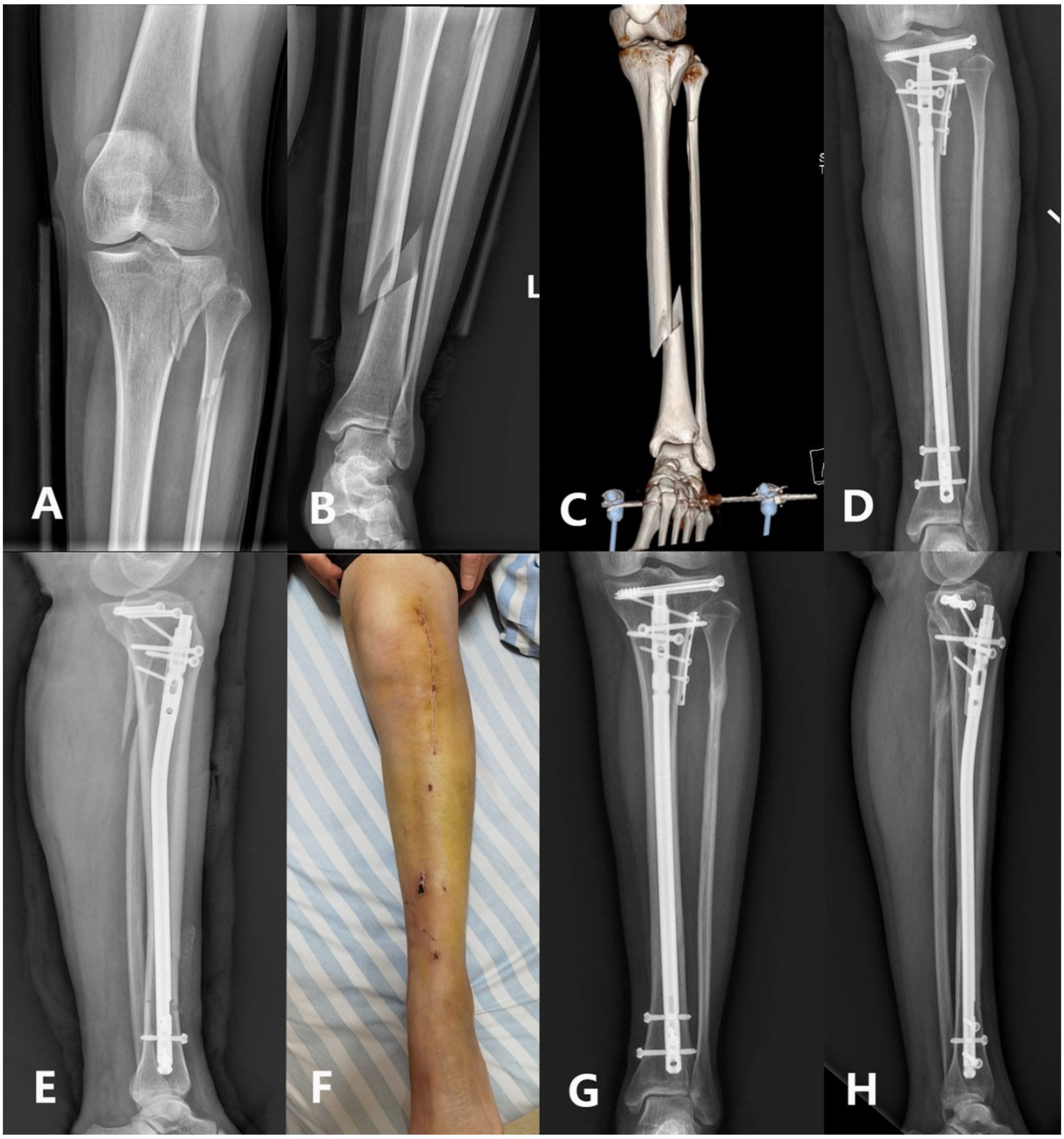

A 42-year-old male suffered a lateral tibial plateau fracture along with ipsilateral distal tibial and fibular fractures on the left leg. The X-ray films (A, B) and three-dimensional CT image (C) demonstrated the morphology of the fractures. He underwent open reduction and internal fixation for the plateau fracture and tibial nailing through an extended lateral parapatellar approach. The postoperative plain

radiographs (D, E) were taken 1 day after surgery. There were no signs of surgical site infection detected at 7 days postoperatively (F). The follow-up radiographs showed bony union was achieved at 6 months postoperatively (G, H).
